# Development of an Automated CAD System for Lesion Detection in DCE-MRI

**DOI:** 10.1007/s10278-025-01445-2

**Published:** 2025-02-20

**Authors:** Theofilos Andreadis, Konstantinos Chouchos, Nikolaos Courcoutsakis, Ioannis Seimenis, Dimitrios Koulouriotis

**Affiliations:** 1 Department of Production and Management Engineering, Democritus University of Thrace, Xanthi, Greece; 2 School of Medicine, Democritus University of Thrace, Alexandroupolis, Greece; 3 School of Medicine, National and Kapodistrian University of Athens, Athens, Greece; 4 School of Mechanical Engineering, National Technical University of Athens, Athens, Greece

**Keywords:** Computer-aided diagnosis, Breast lesions, DCE-MRI, Supervised classification, FFBNP, SVM

## Abstract

Dynamic contrast-enhanced magnetic resonance imaging (DCE-MRI) has been recognized as an effective tool for early detection and characterization of breast lesions. This study proposes an automated computer-aided diagnosis (CAD) system to facilitate lesion detection in DCE-MRI. The system initially identifies and crops the breast tissue reducing the processed image region and, thus, resulting in lower computational burden. Then, Otsu’s multilevel thresholding method is applied to detect and segment the suspicious regions of interest (ROIs), considering the dynamic enhancement changes across two post-contrast sequential phases. After segmentation, a two-stage false positive reduction process is applied. A rule-based stage is first applied, followed by the segmentation of control ROIs in the contralateral breast. A feature vector is then extracted from all ROIs and supervised classification is implemented using two classifiers (feed-forward backpropagation neural network (FFBPN) and support vector machine (SVM)). A dataset of 52 DCE-MRI exams was used for assessing the performance of the system in terms of accuracy, sensitivity, specificity, and precision. A total of 138 enhancing lesions were identified by an experienced radiologist and corresponded to CAD-detected ROIs. The system’s overall sensitivity was 83% when the FFBPN classifier was used and 92% when the SVM was applied. Moreover, the calculated area under curve for the SVM classifier was 0.95. Both employed classifiers exhibited high performance in identifying enhancing lesions and in differentiating them from healthy parenchyma. Current results suggest that the employment of a CAD system can expedite lesion detection in DCE-MRI images and, therefore, further research over larger datasets is warranted.

## Introduction

Breast cancer is the most frequent type of cancer in women and is one leading cause of women’s mortality worldwide [1]. More women die from breast cancer every year than any other type of cancer [2]. In the USA, it is estimated that 12.4% of women will be diagnosed with breast cancer at some point during their lifetime [3]. In addition to that, according to epidemiological studies, the rise of breast cancer cases is anticipated to reach 2 million by the year 2030 globally [4]. The survival rate of patients with breast cancer is low in underdeveloped and developing countries, but according to the literature, there is a great chance of cure on early detection of breast masses via screening, which is an important step in preventing from further progression and the treatment of this disease [5–7].

The most commonly utilized modalities for examining breast lesions and diagnosing breast cancer at an early stage are mammography and ultrasonography due to their fast acquisition, cost-effectiveness, and exploitation facility. However, both approaches sometimes lack essential details needed to recognize the tumour under investigation. More specifically, the evaluation of a mammographic image is subjective and requires a lot of experience as well as its sensitivity can be limited in dense breast tissue due to the presence of overlapping fibroglandular tissue, which reduces conspicuity of abnormalities [8, 9]. Moreover, ultrasonography also requires high analysis expertise due to low image quality [10].

Breast magnetic resonance imaging (MRI) is another non-invasive effective method for detection and characterization of breast cancer, assessment of the local extent of the disease, and guidance for biopsy and localization [11]. It generates high-quality cross-sectional images of the breast, providing a multi-view visual representation. Compared to mammography and ultrasound, MRI has higher sensitivity for detecting breast cancer but is less cost-effective. It has also been demonstrated that occult breast cancers that were not detectable in mammography or ultrasound could be found using MRI [12]. Moreover, MRI has been proven to be a better screening method, especially in women with an increased risk of breast cancer as well as the European Society of Breast Imaging (EUSOBI) recommends the use of breast MRI as supplemental screening to women with extremely dense breasts [13–15]. In addition, MRI supports a number of sub-sequences like dynamic contrast-enhanced MRI (DCE-MRI) which has been used in many recent studies. In DCE-MRI, a paramagnetic contrast agent (Gd-DTPA) is administered intravenously which causes images with relatively high intensity in the tumour region [16]. Although the use of DCE-MRI provides a great opportunity to develop alternative ways to diagnose breast cancer using computer-aided techniques, the evaluation of the enormous amount of images produced for each patient is a time-consuming and error-prone process, and it also depends on the radiologist’s experience [17].

In general, lesion detection is a demanding process due to the variety of sizes and shapes, as well as the low contrast of the respective image segments [18, 19]. Lesions may be oval-shaped or have an irregular-thorny shape with well-defined or ill-defined borders. They may also be associated with glandular elements of the breast, which makes it more difficult to identify. Furthermore, healthy breast tissue and lesions may have similar representation [20]. The simpler way to achieve this task is the radiologists to manually annotate the lesion regions, but as mentioned before, this is a time-consuming and error-prone process which in some cases may lead to an incorrect initial estimation. Therefore, automating this challenging task will help radiologists to obtain more accurate lesion segmentation avoiding the high manual workload. Machine learning has been used in a variety of applications, and several studies have succeeded in separating benign and dangerous cancerous tissue. This study was conducted using machine learning methods in order to detect lesions in breast DCE-MRI images.

## Literature Review

Automated detection of breast cancer in mammography using computer algorithms is a decade-old topic of research, development, and clinical use. With the development of computer-aided diagnosis (CAD) systems, AI has accomplished outstanding performances in medical breast imaging for pattern recognition, object detection (segmentation) and tumour classification as benign or malignant [21, 22]. Object detection, detecting instances of semantic objects of a certain class in digital images, has been applied successfully in conventional X-ray mammography image diagnosis, and nowadays, research focuses on similar techniques to aid or even automatize the diagnosis in MRI of the breast [23].

CAD systems are advanced decision support tools to assist radiologists, providing subjective metrics or visualization of patients’ condition, attempting to improve their diagnosis. They can mark suspicious locations of the breast avoiding overlooked or misinterpreted lesions and reduce the analysis time. Despite though the extended research, there is still room for improvement in the sensitivity and specificity of CAD systems. Research has also shown that a check from a second physician may increase the sensitivity of the method.

CAD systems for mammography using traditional machine learning artificial intelligence methods have been available since the 1990s [24]. Li et al. [25] developed a tumour segmentation method based on adaptive thresholding and a multiresolution Markov random field (MRF) model. The segmented regions of interest were classified into suspicious and normal tissue by a fuzzy binary decision tree. The algorithm was tested on a set of normal and abnormal mammograms achieving 90% sensitivity with two false positive detections per image. Petrick et al. [26] proposed the use of a two-stage adaptive density-weighted contrast enhancement filter in conjunction with a Laplacian-of-Gaussian edge detector for the detection of suspicious mass regions in digitized mammograms. A dataset of 25 mammograms was used, including 11 malignant and 14 benign masses. Using a set of morphological features, they achieved a 96% detection accuracy with 4.5 false positive detections per image. Li et al. [27] introduced a system for mass detection in digitized mammograms based on texture features. They used the bilateral comparison to detect the masses and locate the centre of the regions of interest. Fractal dimension and two-dimensional entropy were calculated as the texture features. Then, the ROIs were classified as masses or normal breast tissue using a support vector machine (SVM). The method achieved a sensitivity of 85.11% with 1.44 false positives per image.

Rejani and Thamarai [28] presented a CAD system that followed a hierarchical scheme of (a) mammogram enhancement, (b) tumour area segmentation, (c) feature extraction, and (d) tumour classification. For the mammogram enhancement, they applied a Gaussian smoothing filter, morphological top hat filtering for eliminating the background, and a discrete wavelet transform (DWT). The tumour region segmentation was implemented using a thresholding technique with different values. Then, morphological features were extracted from the segmented regions, and a SVM was used for the tumour classification achieving a sensitivity of 88.75%. Another system that followed a hierarchical scheme but utilized a two-stage false positive reduction process was developed by Chu et al. [29]. It consisted of five major steps: (1) pre-processing using morphological enhancement which enhanced mass-like patterns, (2) segmentation based on a simple linear iterative clustering (SLIC) method, (3) pre-screening of suspicious regions using a rule-based classification, (4) potential lesion contour refinement based on distance regularized level set evolution, and (5) false positive reduction based on feature extraction and SVM classification. For the evaluation of the system, 474 digitized mammograms were used, divided into two datasets. Two-thirds of the available masses were used in the training set, and the remaining masses and normal breast tissue regions were used in the test set achieving 81.4% accuracy.

Many studies have also been tested on publicly available datasets, providing similar results. Mughal et al. [30] used texture and colour features to detect and classify masses in mammograms. They applied contrast limited adaptive histogram equalization (CLAHE) for enhancing the contrast of the mammogram as well as mean filter and wavelet transformation to reduce the image noise. A segmentation method composed of two phases was introduced for the extraction of the breast region, followed by a mathematical morphology function which extracted and refined the ROIs. Texture and morphological features were extracted and used for classification, where different classifiers (SVM, decision tree, KNN) were applied. The SVM having a quadratic kernel had the best performance on the DDSM and the MIAS datasets. Punitha et al. [31] proposed an automated detection method of masses in mammograms. They used Gaussian filtering for smoothening the grey level variations and reducing the noise in the image. Then, an optimized region growing technique was applied, where the initial seed points and thresholds were optimally generated using a dragonfly optimization (DFO). A set of 45 texture features was extracted using Gray Level Co-occurrence Matrix (GLCM) and Gray Level Run Length Matrix (GLRLM) techniques and fed into a feed forward back-propagation neural network for classification. The method was evaluated using images obtained from the DDSM database where sensitivity and specificity reached up to 98.1% and 97.8%, respectively. The CAD model presented in the work of Mohanty et al. [32] was also validated on the MIAS and the DDSM datasets. It utilized a discrete Tchebichef transform (DTT) to extract the features from the ROIs, followed by a combination of principal component analysis (PCA) and linear discriminant analysis (LDA) which was reducing the dimension of each feature vector. Finally, the reduced features were classified using an extreme learning machine (ELM) achieving 100% and 99.5% accuracy for each dataset, respectively.

In the last decade, more studies focused on deep learning methods for breast lesion segmentation and diagnosis because, in contrast to traditional machine learning, deep learning methods combine feature extraction and model training into a single cohesive learning framework so that the segmentation task is performed in an end-to-end manner. Dhungel et al. [33] presented an approach for the detection of masses in mammograms that combined a cascade of deep learning and random forest classifiers. The first stage classifier contained a multi-scale deep belief network (m-DBN) that selected the suspicious regions which were further processed by a two-level cascade of deep convolutional neural networks (CNNs). Texture and morphological features were extracted from the regions that survived the first stage classifier, in order to be classified by a two-level cascade of random forest classifiers. The system was tested using two publicly available datasets, the INbreast and the DDSM-BCRP. A fivefold cross-validation per patient was applied, achieving 96% sensitivity with 1.2 false positive per image on the INbreast dataset, and 75% sensitivity with 4.8 false positive per image on the DDSM-BCRP dataset. In the work of Al-antari et al. [34], a deep belief network-based CAD system was proposed. For the detection of the initial suspicious regions, they used an adaptive thresholding method followed by consecutive morphological operations. Two techniques were utilized to extract the ROIs: in the first, four non-overlapped ROIs were extracted randomly from each detected region, whilst in the second, the whole detected region was used. Texture, morphological, and statistical features were calculated and used in classification. Different classifiers, namely linear discriminant analysis (LDA), quadratic discriminant analysis (QDA), neural network (NN), and deep belief network (DBN), were applied. The results showed that DBN outperformed the other classifiers. Ribli et al. [35] adopted the object detection framework Faster R-CNN to build a system that detects and classifies malignant or benign lesions in mammograms. The DDSM database was used for the training of the system whilst the INbreast database for testing. Due to the low quality of the digitized film-screen mammograms, they mapped the pixel values to optical density using calibration functions and then rescaled the pixel values to the 0–255 range. The base CNN used was a VGG16 network which is a 16-layer deep CNN. Its final layer classified the lesions into benign or malignant providing a confidence score that indicated the respective class. The system achieved an AUC of 0.95 for classification and was able to detect malignant lesions with a 90% sensitivity with 0.3 false positive marks per image.

Although several CAD systems utilizing different breast imaging techniques have been developed for the detection and diagnosis of breast lesions, only a limited number of studies deal with the automatic detection of lesions in breast DCE-MRI. In the work of Koper et al. [36], three breast lesion segmentation algorithms were compared using the publicly available QIN Breast DCE-MRI database. In their approach, the breast lesion segmentation algorithm consisted of two main steps. In the first step, the approximate breast mask was obtained by subtracting the first image in a series of subsequent MRI images, reducing the search area and the number of false positive. In the second step, the breast lesions within the received breast mask were segmented. During the second step, they tested a threshold-based method using the classic Otsu algorithm, a seeded region growing method where the initial seeds were picked manually by the user, and a clustering method using the *K*-Means algorithm having four clusters. In each case post-processing was performed based on morphological operations. The evaluation of the segmentation methods was done using Jaccard’s Similarity Coefficient and Dice’s Similarity Coefficient which both obtained better results for the K-Means clustering method. Khaled et al. [37] used a modified U-Net framework, combining three U-Net models each one using a different combination of input volumes, for automatic breast lesions segmentation from DCE-MRI. Individual results of each model are combined into a single segmentation by a simple union operation of the segmentations. In their method, a ROI restricted balanced patch extraction has been utilized, in order to address both the class imbalance and confounding organs problems. The system’s evaluation was done with the use of a subset of 46 cases from the TCGA-BRCA dataset, obtaining a mean Dice similarity coefficient (DSC) of 0.802 for the main lesions, and 0.680 for the total lesions.

In the recent work of Haq et al. [38], an automatic breast tumour segmentation process was presented using conditional GAN (cGAN). They used a U-NET model architecture as a generator network having a parallel dilated convolution (PDC) module between an encoder and a decoder in order to recognize breast tumour-related features of various sizes of masses, and extract information about masses’ edges and interior texture. Then a Patch-GAN discriminator was used to produce the probability score and decide if the input image is genuine or fake, based on known ground truth (binary mask) images. Another automatic approach for DCE-MRI breast lesion segmentation was introduced in the work of Jiadong et al. [39]. More specifically, they proposed an artificial intelligence (AI) assistant for breast tumour segmentation in multi-phase DCE-MRI, by capturing dynamic changes across multiple phases with a spatial–temporal framework. They developed a whole-breast segmentation model, adopting a U-Net structure, in order to localize the breast region and ensure that the segmentation model focused solely on that area. Both the original and the subtraction images were used as input, and the dynamic spatial–temporal information was captured via a spatial–temporal transformer. Segmentation results indicated a good agreement between the proposed model and the manual annotations by radiologists proving the model’s robustness and efficiency.

Similar studies have focused not only on the segmentation but also the classification of the segmented suspect areas, providing a more complete diagnostic tool for DCE-MRI. The work of Zheng et al. [40] proposed a framework for accurate characterization of tumours in contrast enhanced MR images. They developed a graph cut-based segmentation algorithm to improve coarse manual segmentation, allowing precise identification of tumour regions. In order to initialize the segmentation algorithm, tumours were specified and roughly segmented by a manual rater. Then, by considering serial contrast-enhanced images as a single spatiotemporal image, a spatiotemporal model of segmented tumours is constructed to extract spatial and temporal enhancement features using a discrete Fourier transform (DFT) and via moment invariants and rotation invariant Gabor textures. The experimental results showed that the proposed framework had high accuracy, yielding a value of 0.97 for the area under the receiver operating characteristic curve. Rasti et al. [41] presented a novel CAD system for breast DCE-MRI using a mixture ensemble of convolutional neural networks (ME-CNN) to discriminate between benign and malignant breast tumours. The proposed system had two major stages. In the first stage, contrast enhancement and breast region cropping were employed, allowing the candidate tumours to be segmented based on the intensity and morphological information of the masses. In the second stage, the selected tumours were classified based on the ME-CNN model composed of three CNN experts and one convolutional gating network, achieving an accuracy of 96.39%, a sensitivity of 97.73%, and a specificity of 94.87%. Shokouhi et al. [42] implemented an automated CAD system for the detection of breast lesions in DCE-MRI using a seeded region growing algorithm based on Fuzzy C-means clustering and the vesselness filter. The proposed system initially segmented the breast region and then used the detected potential lesion voxels as the initial seed points of the region-growing algorithm. Subsequently, morphological and kinetic features were calculated and fed in a SVM classifier in order to reduce the false positive lesion regions.

Adachi et al. [21] built and evaluated an AI system to detect and diagnose lesions of maximum intensity projection (MIP) in breast DCE-MRI. Breast lesions were indicated by a radiologist, whilst the AI system was trained to detect and calculate the possibility of malignancy using RetinaNet. The system showed better diagnostic performance compared to four human readers who also scored the same data set, regarding the possibility of a malignancy in each breast, with and without the assistance of the developed AI system. In the work of Hu et al. [43], a deep transfer learning computer-aided diagnosis methodology was developed to diagnose breast cancer using multiparametric MRI. Each MR study included a DCE-MRI sequence and a T2-weighted MRI sequence. A pretrained convolutional neural network (CNN) was used to extract features from the DCE and T2w sequences, and SVM classifiers were trained on the CNN features to distinguish between benign and malignant lesions. The classification results showed that the use of a multiparametric scheme incorporating the complementary information provided in DCE and T2w MRI sequences outperformed the two single-sequence-based methods.

The main goal of the current study is to develop an automated CAD system to facilitate breast lesion detection in DCE-MRI, combining existing as well as newly implemented techniques. Specifically, a novel breast region segmentation method is introduced, which calculates the cropping window using the intensity values of the image pixels, combined with image rotations, and without the use of complex algorithms that may negatively affect execution time as well as the system’s complexity. Furthermore, the implemented segmentation algorithm detects and segments the suspicious regions of interest (ROIs) considering the dynamic enhancement changes across two post-contrast sequential phases, thus differentiating the current work from similar previous studies. In addition, the proposed approach of creating the control ROI group attempts to address the challenging problem of small datasets in single-centre imaging studies. For the evaluation of the system, two classifiers widely used in medical imaging research were employed.

## Materials and Methods

### Dataset

The DCE-MRI data used in this study were collected at the University General Hospital of Alexandroupolis between September 2018 and August 2023. The imaging data were fully anonymized and processed according to the institutional ethical standards. All provisions of the General Regulation (2016/679) of the European Parliament and the relevant national legislation (law 4624/2019) on the protection of personal data and the preservation of the anonymity and confidentiality have been complied with. Patients were selected based on the available radiologists’ reports in picture archiving and communication system (PACS). The images and any other patient data were anonymized by globally altering all the relevant DICOM tags in the imaging dataset. This was accomplished by using the anonymize option during the export process via the PACS system.

The examinations were performed in the prone position on a Philips Multiva 1.5 T MRI system using a 16-channel breast coil. Imaging was performed prior to and after a bolus injection of Gd-DTPA (0.2 mL/kg) in intravenous way. The first sequence was taken before the agents’ injection, followed by six post-contrast series which were obtained approximately every 70 s. Each patient DCE-MRI series was provided as a DICOM file that combines all pre- and post-contrast volumes as different channels. Image analysis was performed using an inhouse developed script written in the MATLAB programming language (The MathWorks Inc.). In total, 119 DCE-MRI slices were obtained having 138 histopathologically proven enhancing lesions (64 malignant and 74 benign). The patients’ age ranged from 30 to 77 years.

### Image Segmentation and ROI Selection

**Fig. 1:**
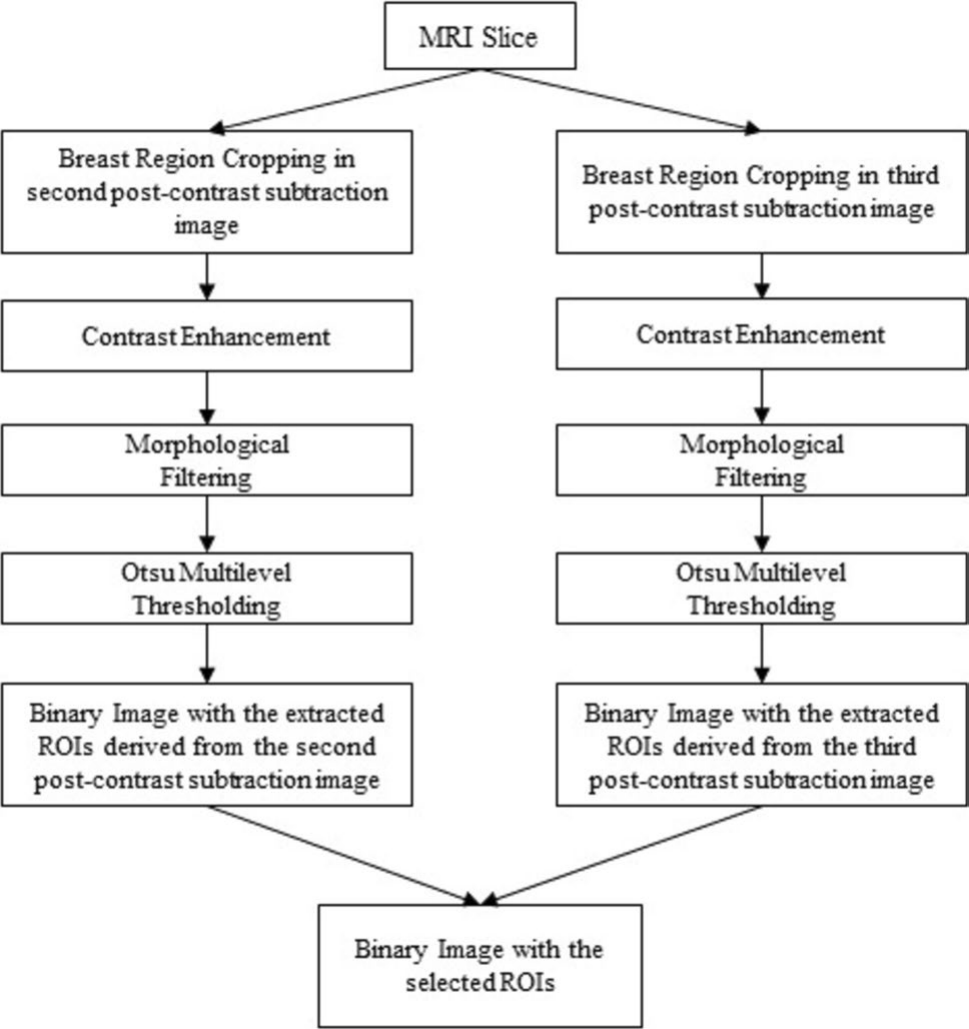
Image segmentation and ROI selection

Based on the available radiologists’ reports, a set of breast axial slices was selected in order to represent the best appearance of the lesions amongst other image slices. In Fig. 1, the main steps of the automatic image segmentation and ROIs selection algorithm are presented. For each selected slice, the breast region was identified and cropped in the second and third post-contrast subtraction image, followed by contrast enhancement and morphological filtering. The selected ROIs were obtained after the application of Otsu’s multilevel image thresholding [44] and the intersection of the created binary images.

#### Breast Region Segmentation

The DCE-MRI slices include confounding backgrounds such as internal vital organs (heart and lung area), vessel structures, and anatomical structures (chest area). These areas have similar imaging with lesions, since they have high changes in their intensity values across the time after the contrast injection, making it harder to detect the lesions, and lead to increased false positive findings. Therefore, breast region segmentation is a very important step that significantly improves tumour segmentation performance, and this is why it has been exploited in many studies in literature [39, 45].

**Fig. 2:**
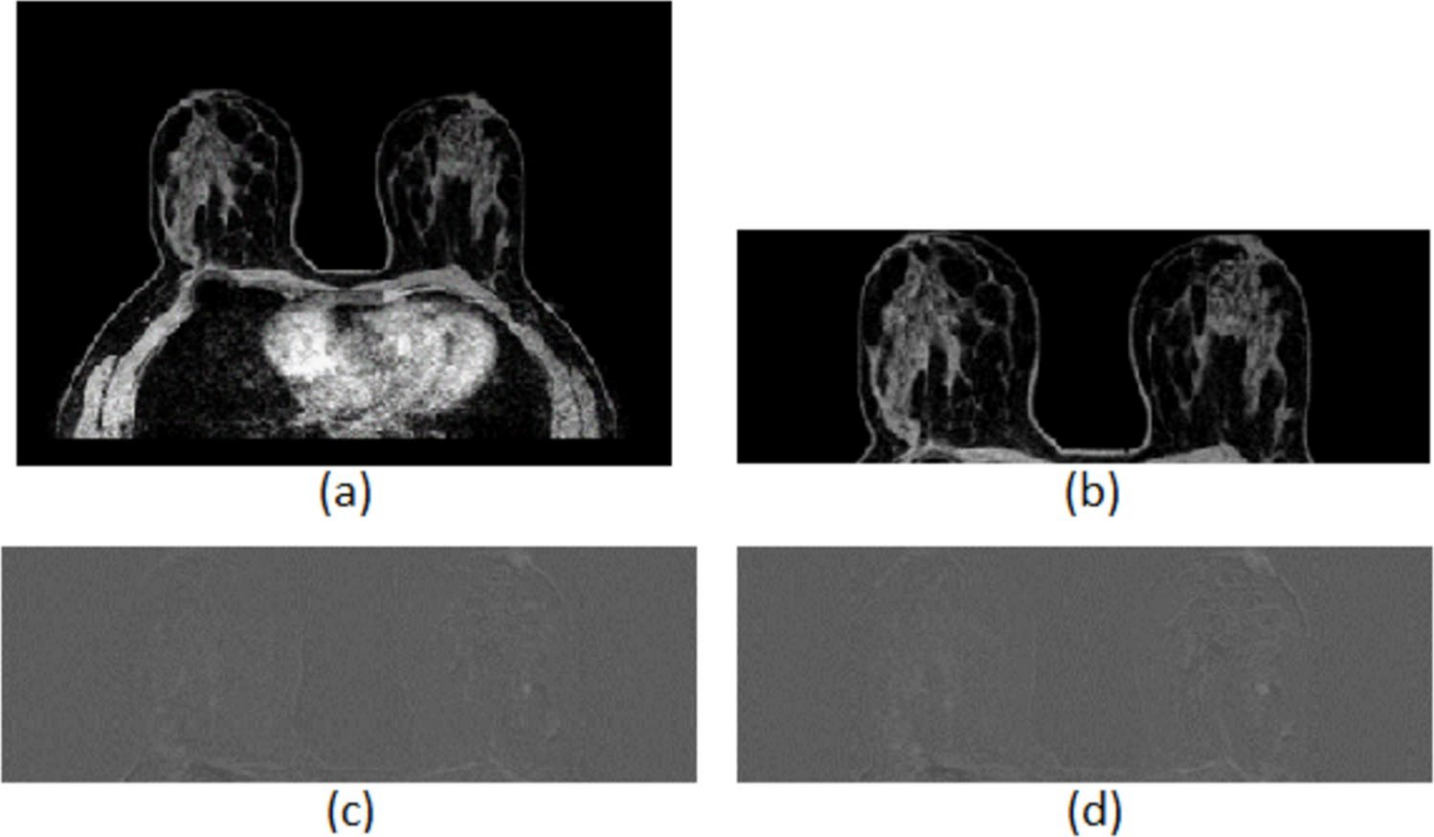
Breast region segmentation: **a** initial MRI slice, **b** breast area in the initial MRI slice, **c** breast area in the second post-contrast subtraction image, and **d** breast area in the third post-contrast subtraction image

In this paper, we implemented an automatic method to identify the approximate size and location of the breast tissue, reducing the processed image region and, thus, resulting in lower computational burden. The lower computational burden is determined compared to the significantly additional execution time that would be required if the automated breast region segmentation technique was not used, and therefore, the search area would not have been reduced to a smaller region. The size and position of the rectangular cropping window was determined by the following steps:

- We identify the minimum and the maximum value of the *Y* coordinate, *Y*_min_ and *Y*_max_, that corresponds to the pixels whose intensity value is greater than the background.
- We identify the minimum value of the *X* coordinate, *X*_min_, that corresponds to the pixel whose intensity value is greater than the background.
- We identify the minimum value of the *X* coordinate that corresponds to the pixel whose intensity value is greater than the background in the median column of the image *Y*_middle_. This value is defined as *X*_max_ and is being increased by 10 pixels, corresponding to 6.5 mm, in order to reassure that the cropping window will properly include both breasts in case that one breast is bigger than the other.
- We crop the initial image, which is obtained before the administration of the contrast agent (Phase 0), as well as the second and third post-contrast subtraction images according to the calculated cropping window. The window has as top corner the position (*X*_min_, *Y*_min_), width the distance *Y*_max_—*Y*_min_, and height the distance *X*_max_—*X*_min_. An example is shown in Fig. 2.

#### Image Pre-processing

After the extraction of the breast area, the following steps were applied both in second and third post-contrast subtraction images, as a pre-processing stage in order to reduce image noise:

- Application of contrast limited adaptive histogram equalisation (CLAHE) using exponential distribution [46]. Considering *I*_*s*_ as the cropped post-contrast subtraction image, the corresponding enhanced image *I*_*enh*_ was computed according to MATLAB’s function for CLAHE technique.
  1 $${I}_{enh}=\text{adapthisteq }\left({I}_{s}\right)$$
- Morphological filtering using an alternating sequential filter (ASF) which consists of two sequential morphological openings and closings, using disks of increasing radius as structural elements (se). The filter was applied to *I*_*enh*_ computed in the previous step, and the generated image was called *I*_*flt*_.

**Fig. 3:**
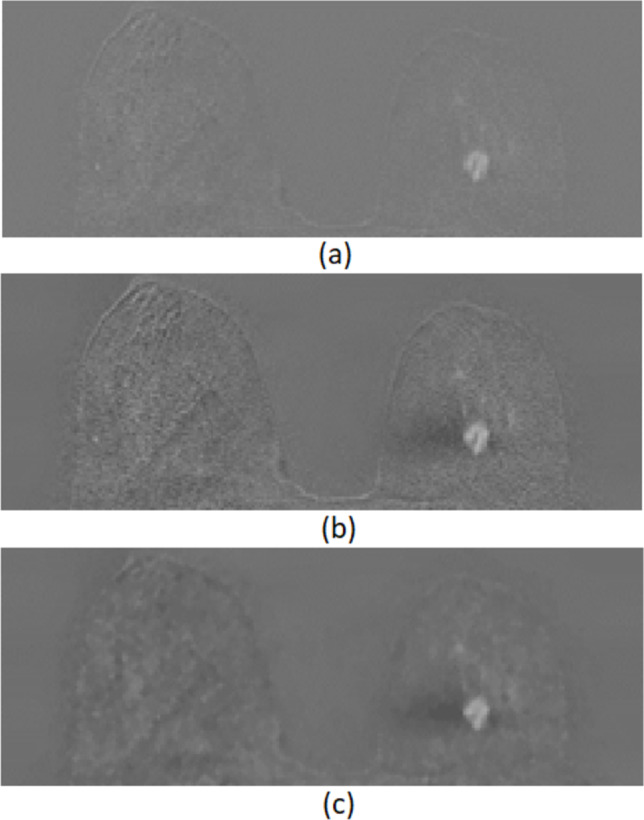
Image pre-processing: **a** cropped post-contrast subtraction image *I*_*s*_, **b** enhanced image *I*_*enh*_,, and **c** image after the ASF application *I*_*flt*_

The image pre-processing steps described above are presented in Fig. 3.

#### Lesion Detection

Contrast enhancement of breast lesions shows physiologic variations, mostly due to differences in vascular permeability. Differences may also depend on lesion histology or on inhomogeneities within the lesions [47]. Moreover, studies have demonstrated that in DCE-MRI, both benign and malignant lesions absorb the contrast agent in the early phases and can be discriminated based on its release over time using the time-signal intensity curve (TIC) [48]. TIC is obtained for each region of interest, and it differs significantly for benign and malignant enhancing lesions. Specifically, plateau and washout patterns are more likely associated with cancerous tissue, whereas persistent pattern is a typical form of benign enhancing lesions [49, 50].

In our study, the second and third post-contrast subtraction images were used, including in the segmentation process only regions that presented contrast enhancement in both time-points. Otsu’s multilevel image thresholding was applied for the extraction of all suspicious areas on the basis of contrast enhancement. In particular, the following steps were applied for each axial MRI slice:

1. The automatic segmentation method was applied on the second post-contrast subtraction image. The new image created, *I*_*s*2_, contained only the corresponding breast area.
2. The pre-processing steps, CLAHE and morphological filtering, were sequentially applied on image *I*_*s*2_. The generated filtered image was named *I*_*ftl*2_.
3. Otsu’s multilevel thresholding method was applied on *I*_*flt*2_. Initially, four distinct threshold levels were calculated, dividing the image into five intensity value classes, and then multilevel thresholding was applied. The result was a binary image, *I*_*otsu*2_, containing only the regions corresponding to the class with the highest intensity value. These are considered to be the enhancing ROIs derived from the second post-contrast subtraction image.
4. The process described in Steps 1–3 was repeated for the third post-contrast subtraction image. The corresponding binary image created, *I*_*otsu*3_, contained the enhancing ROIs derived from the third post-contrast subtraction image.
5. The image containing the suspicious (i.e. enhancing) regions of interest for the specific MRI slice, *I*_*otsu*_, was calculated by the intersection of the two binary images *I*_*otsu*2_ and *I*_*otsu*3_: *I*_*otsu*_ = *I*_*otsu*2_ ∩ *I*_*otsu*3_

**Fig. 4:**
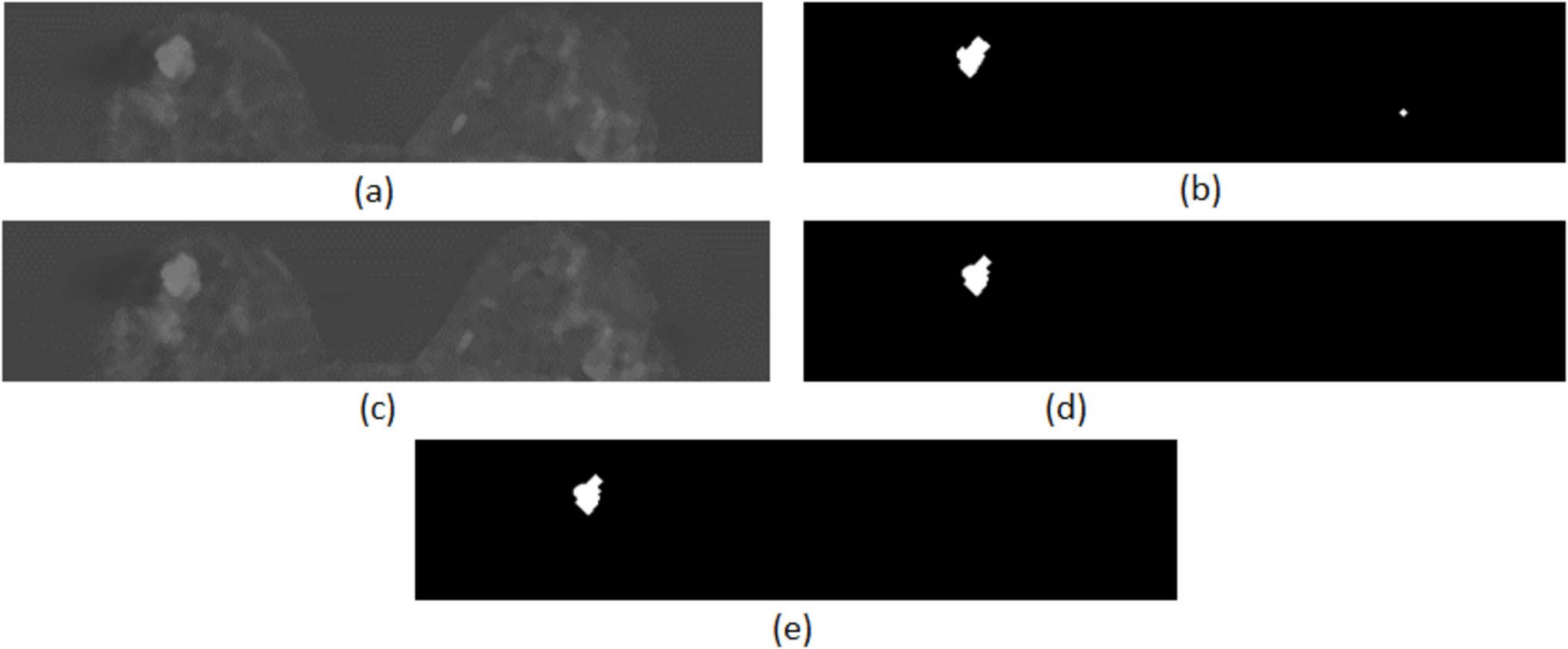
Initial ROI extraction in an MRI slice: **a** image *I*_*flt*2_, **b** binary image *I*_*otsu*2_, **c** image *I*_*flt*3_, **d** binary image *I*_*otsu*3_, and **e** initial ROI extraction for the MRI slice by intersection of the two generated binary images—image *I*_*otsu*_

**Fig. 5:**
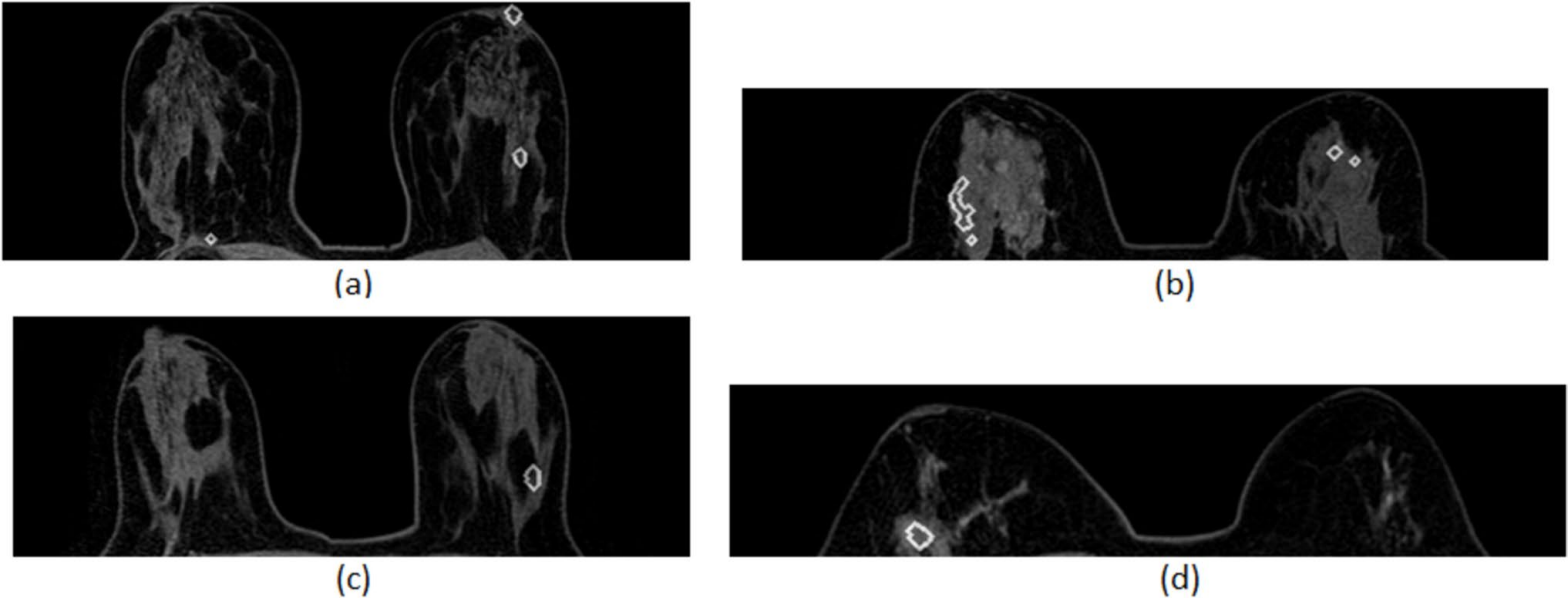
Segmentation results in MRI slices: **a, b** segmentation results in cases with benign lesions and **c, d** segmentation results in cases with malignant lesions

The proposed segmentation method extracts the initial ROIs taking into consideration the dynamic changes across two sequential phases. Furthermore, it is estimated that it may successfully detect lesions regardless of the breast type (fatty-glandular or fatty). However, some of the derived suspicious areas correspond to healthy breast tissue. The segmentation process and some segmentation results are depicted in Figs. 4 and 5.

### False Positive Reduction Process

**Fig. 6:**
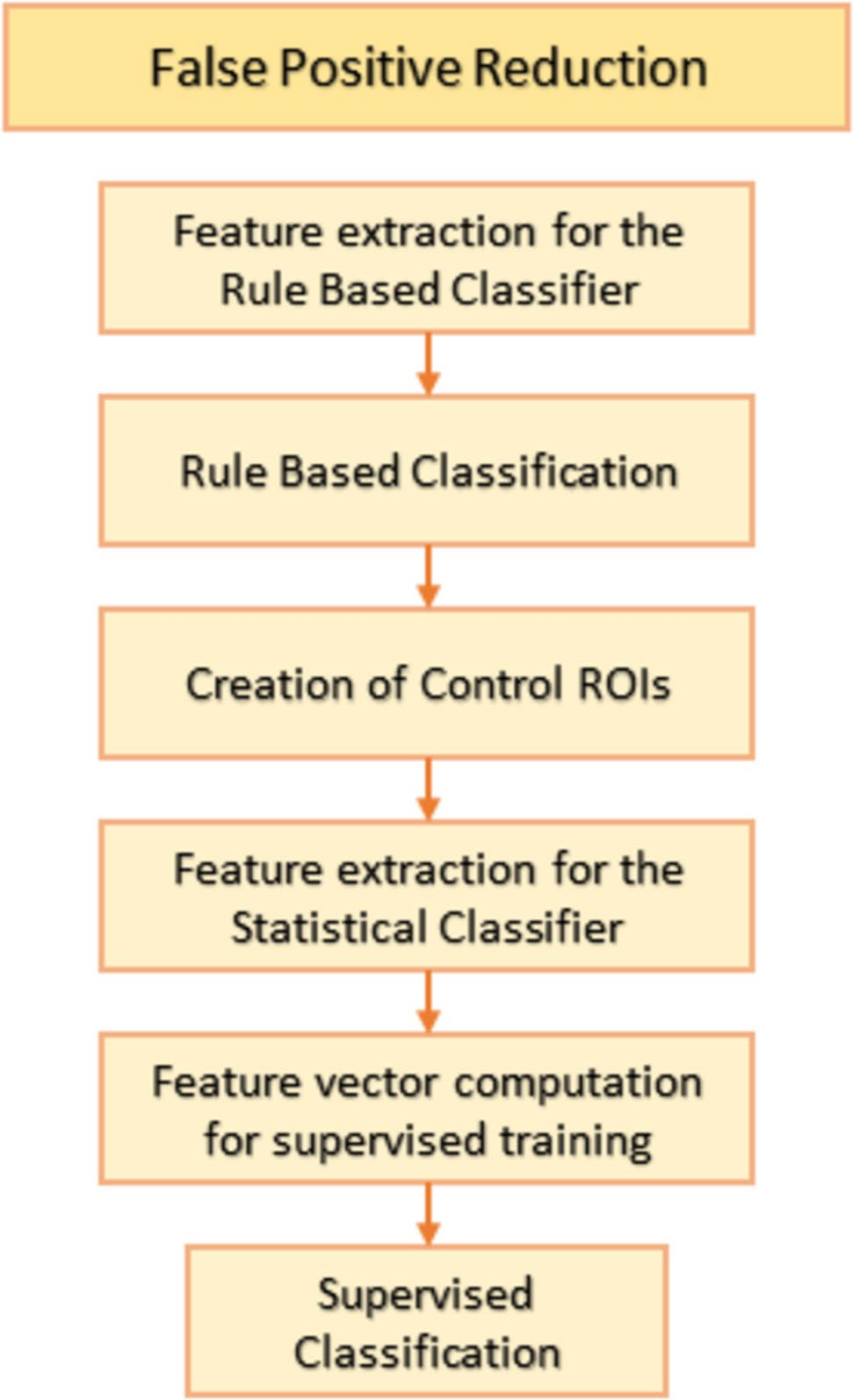
False positive reduction process

Even though in the previous stage only the suspicious areas detected in both post-contrast phases were extracted, these regions do not necessarily correspond to malignant or benign lesions, but they may also represent healthy breast structures (e.g. vessels). These false detections can affect the performance of the CAD system. Classifiers may be used to reduce the number of false positive detections, a process called false positive reduction. In this work, a two-stage classifier was implemented, since it enables the combination of different methods in the false positive reduction process. In particular, a rule-based classifier which rejects suspicious regions of interest using geometric features is initially applied, followed by supervised classification techniques that differentiate lesions from healthy parenchyma. The proposed technique has been successfully used in other similar studies [29, 33, 51]. The false positive reduction process is shown schematically in Fig. 6.

#### First Classification Stage

At this stage, a simple rule-based classifier was applied. It rejects suspicious areas with dimension smaller than a minimum size or have high eccentricity values. Specifically, a suspicious area was rejected if:

- Its size was less than 15 pixels which corresponds to lesions of 10 mm size, since the resolution of the images is 654.762 μm/px. According to literature, the most common MRI-detectable lesion is a mass, which is a three-dimensional, space-occupying lesion ≥ 5 mm in size (on each axis). However, conventional examinations generally detect masses ≥ 1 cm [52]. Moreover, breast MRI has been found to detect 64–75% of occult breast malignancies, with average tumour size of 15 mm at detection [53]. This approach also avoids the detection of small vessels.
- Its eccentricity value was greater than 0.95. Eccentricity is a morphological feature that specifies the eccentricity of the ellipse that has the same second moments as the lesion region. A perfectly round lesion will have an eccentricity of 0, whilst a lesion that is long and thin will have an eccentricity of 1.0 [54]. In MRI, regions with high eccentricity value may be associated with noise or vessel structures of the breast [55].

**Fig. 7:**
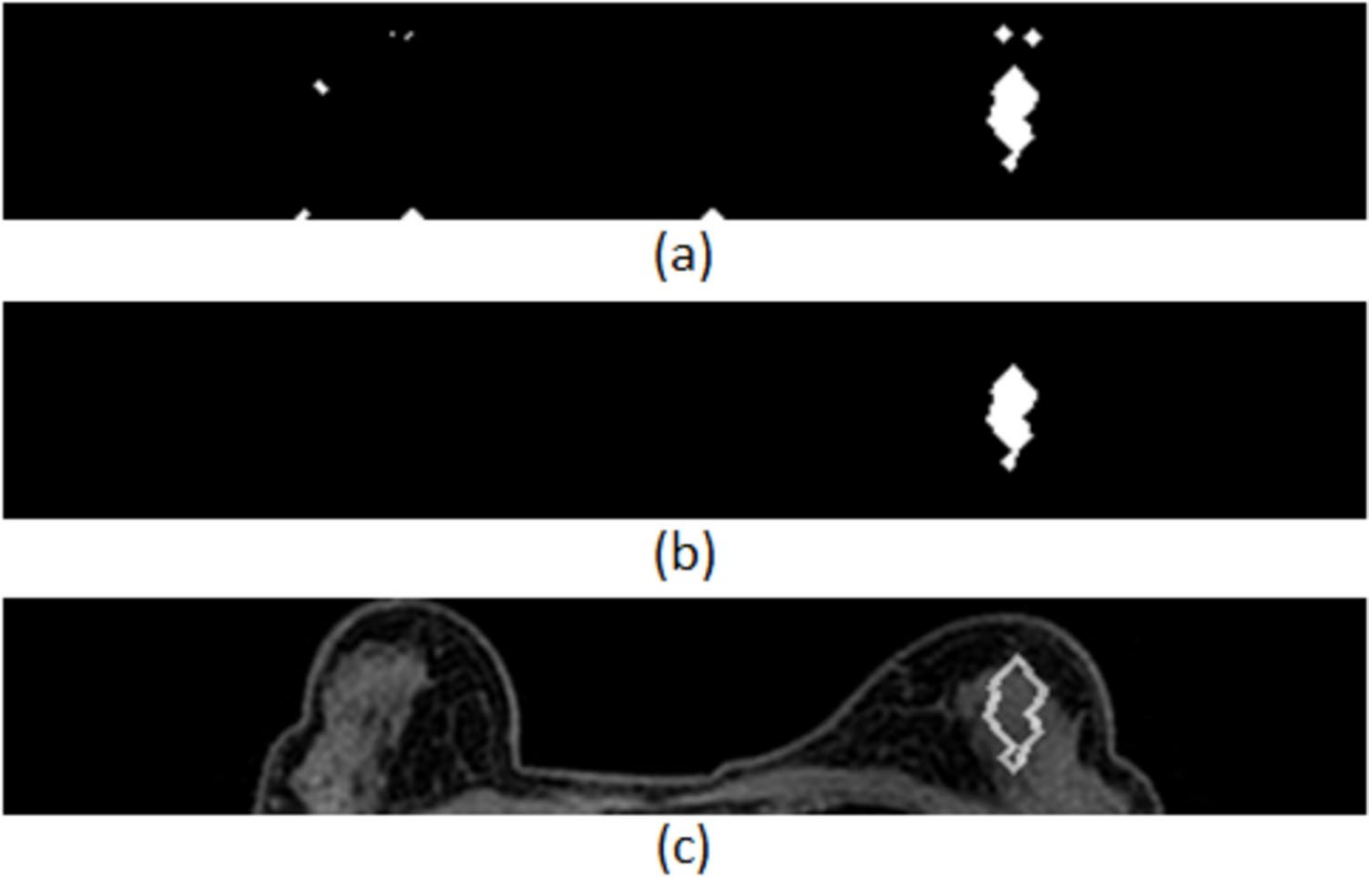
Suspicious areas reduction: **a** initially extracted areas, **b** suspicious areas remained after the application of the first classifier, and **c** outline of the remaining areas overlaid on the original MRI slice

Figure 7 shows the reduction of the suspicious areas after the application of the first classifier.

#### Creation of Control ROIs

**Fig. 8:**
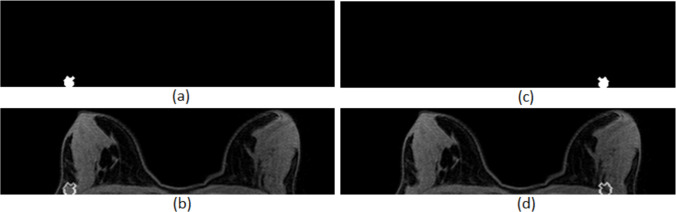
Control ROI creation: **a** extracted ROI, **b** outline of the extracted ROI overlaid on the MRI slice, **c** created control ROI, and **d** outline of the control ROI superimposed on the MRI slice

For the suspicious regions that were not rejected by the first classifier, symmetric ROIs were automatically created on the other breast, using a script implemented in MATLAB software. Specifically, each region of interest was duplicated and mirrored on the contralateral breast, using the vertical axis passing through the centre of the breasts as the axis of symmetry. Furthermore, each symmetric ROI was inverted with respect to the vertical axis that passes through the centre of the symmetric lesion in the anterior–posterior direction, so that its relative position on the underlying breast to correspond to the position of the detected enhancing lesion on the breast with the initially detected lesion. Therefore, the control ROIs had the same shape, the same size (number of pixels), and about the same relative position on the breast (breasts are not identical) with the corresponding original ROIs representing detectable regions. This way equivalency between lesion and control datasets was reassured. The control ROIs were inspected by an experienced radiologist to confirm correspondence and to secure that the control areas did not present any suspicious imaging characteristics. Figure 8 shows an example of a control ROI creation.

#### Feature Extraction

After the creation of the group of the control ROIs, several features were calculated in order to be used in the second classification stage. Features were calculated for both the extracted and the control ROIs, in the image obtained before the administration of the contrast agent (Phase 0). Furthermore, all ROIs (extracted and control) were dilated using a disk radius of 1 pixel as structural element (se), to cover any potential minor patient movement between pre- and post-contrast phases. To avoid considerable increase of the ROI area, the minimum possible size of 1 pixel was chosen as the size of the structuring element, since the examinations were performed in the prone position which is supposed to minimize patient movement. Moreover, the time interval between Phase 0 and the second post-contrast phase, where the ROIs were initially delineated, was approximately 140 s, which is quite small reducing the likelihood of minor patient movement.

##### Texture Features

Texture is represented by the spatial distribution of the gray levels in an image and is one of the important characteristics used in identifying objects or regions of interest in an image [56]. For the texture features extraction, the Gray Level Co-occurrence Matrices (GLCM), also known as gray-level spatial dependence matrices, were used. The GLCM contain the second-order statistical information of spatial relationships of pixels of an image and show how many times pairs of gray level values exist in defined distances and directions. Directions used were 0, 45, 90, and 135 [57]. The selected features should not be affected by the rotation of the objects, such as the mean value and the range between the maximum and the minimum value [56]. The calculated GLCM features were contrast, correlation, energy, homogeneity, entropy, and inverse difference moment which have been used in many related studies of breast DCE-MRI imaging [58–61]. The features were calculated for distances of 1 and 2 pixels (d = 1 and d = 2).

##### Contrast Features

Contrast features were measured as intensity differences between inside and outside of a ROI. A region growing technique was initially applied using a disk radius of 4 pixels as structural element, and subsequently, the ROI was subtracted from the “grown” area, creating a ring. The size of the structuring element was chosen based on the shape properties of the extracted regions of interest. Specifically, since a certain area that needs to be enclosed requires a minimum perimeter, the ratio of the region’s area to its perimeter was calculated for each ROI. Then, the distribution of the ratios for all lesions was examined, and it was noticed that most of the lesions had a ratio close to 4. Thus, the size of the structuring element was arbitrarily set to 4 pixels, allowing however the creation of rings of appropriate area.

A contrast feature according to Dominguez and Nandi [62] is the ratio:

2 $$CON=\frac{{I}_{in}-{I}_{r}}{{I}_{in}+{I}_{r}}$$

where “CON” is the calculated contrast value, *I*_*in*_ is the mean intensity value inside the ROI, and *I*_*r*_ is the mean intensity value inside the created ring around the ROI.

According to Brake and Karssemeijer [51], contrast can also be measured with the creation of a ring around the ROI, equally sized. In particular, contrast is the ratio:

3 $$C=\frac{({I}_{in}-{I}_{r}{)}^{2}}{{\sigma }_{in}^{2}+{\sigma }_{r}^{2}}$$

where “*C*” is the calculated contrast value and *I*_*in*_, *σ*_*in*_, *I*_*r*_, and *σ*_*r*_ are the mean value and the standard deviation of the intensity inside the ROI and the created ring correspondingly. Mean intensity value and standard deviation of the ROI were also used as separate features.

##### Geometric Features

The fact that the group of the control ROIs is symmetric to the extracted suspicious areas, having the same shape, restricts the use of geometric features. Moreover, the proposed CAD system tries to segregate lesions from the healthy breast tissue, without taking in consideration if they are malignant or benign. Usually, the malignant lesions have thorny shapes. However, in many cases, mammographic features are overlapped between malignant and benign lesions [63]. In this paper, the only geometric feature used was eccentricity during the application of the rule-based classifier.

#### Second Classification Stage (Feature Reduction and FFBPN/SVM Application)

Of the 14 features calculated for each ROI, only 8 were selected for the classification stage. The features selection was done using the built-in MATLAB functions, Sequentialfs and Relieff. The Relieff function ranks the features using the ReliefF algorithm in combination with the k-nearest neighbors (KNN) algorithm. The Relief algorithm was proposed by Kira and Rendell in 1992 [64], and ReliefF constitutes its improved version for feature selection. It is a widely used feature selection method and has been proven to be sensitive to complex patterns of association [65, 66]. The algorithm penalises the features that give different values to neighbours of the same class, whilst it rewards features that give different values to neighbours of different classes. The Sequentialfs function selects a subset of the available features, identifying the most relevant for the successful classification. For the elimination of the redundant features, the Recursive Feature Elimination (RFE) algorithm is used, which iteratively removes features based on their contribution to model accuracy [67]. Specifically, the initial feature set included all the calculated features, and Sequentialfs removed one feature from the set at each iteration, until removing a feature did not decrease the criterion function. In the current study, the selected criterion function was the misclassification rate.

In the second classification stage, a feed-forward backpropagation neural network (FFBPN) and a SVM were independently tested for further reduction of the false positive areas, and the evaluation of the proposed CAD system. Both classifiers are supervised classification methods and have been extensively used in biomedical engineering and medical diagnosis. ANNs may provisionally present lower data requirements than other model types. They can be flexibly scaled to any available dataset, and under certain circumstances, they can deal very well with incomplete or missing data [68]. Also, unlike the SVM, extreme learning machine, and random forest models, ANNs are more fault tolerant [69]. On the other hand, SVMs have good performance with unstructured and semi-structured data like images.

**Fig. 9:**
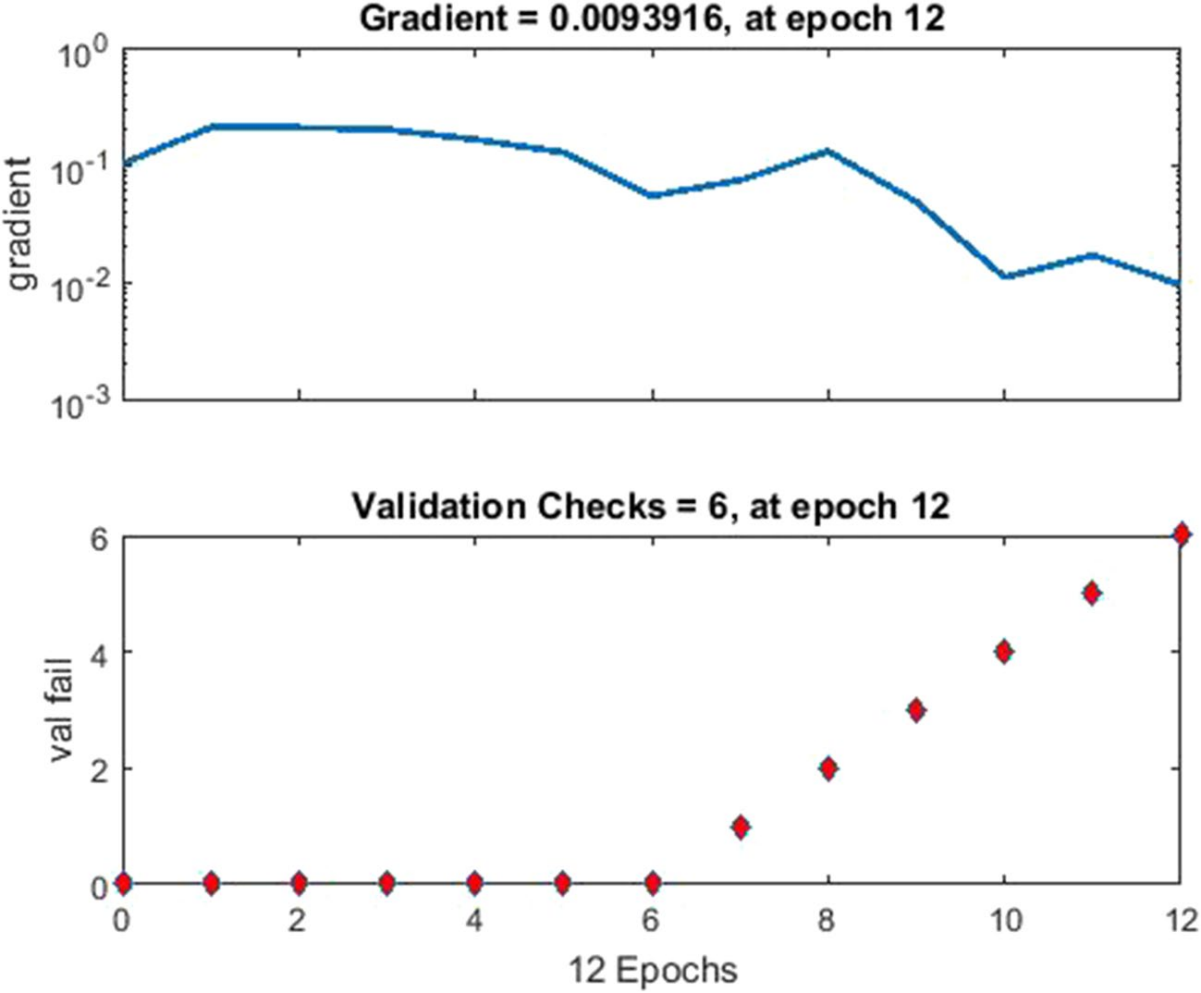
Network training having 12-epoch duration

**Fig. 10:**
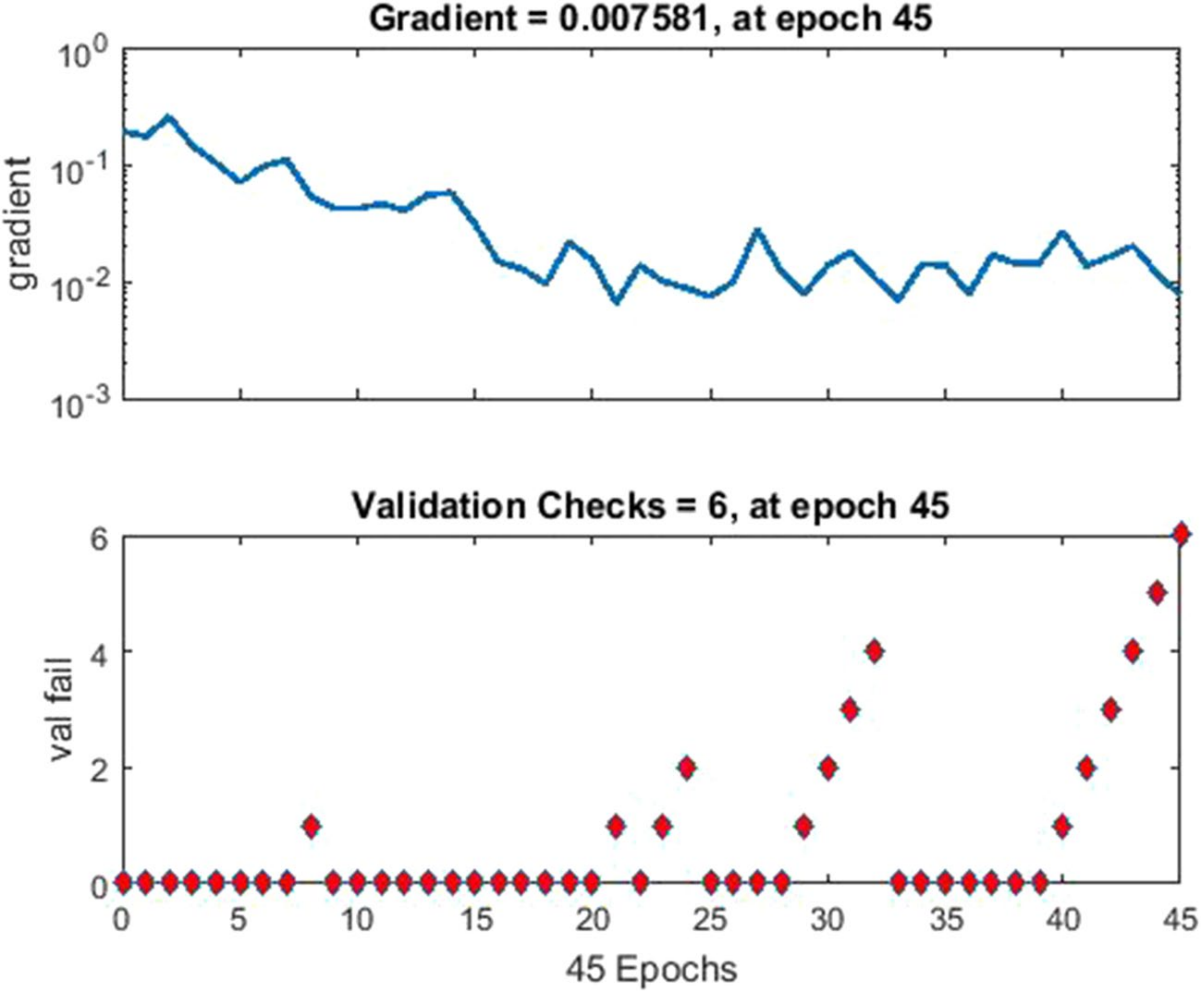
Network training having 45-epoch duration

The ANN used had one hidden layer where different number of neurons was tested, *n* = 6 and *n* = 10. The input layer had eight neurons, one for each feature of the calculated feature vector. The output layer had two neurons since the suspicious areas were classified into two classes: lesion or healthy breast tissue. Scaled conjugate gradient backpropagation (trainscg) was selected as network training function to update weight and bias values, and hyperbolic tangent function (tansig) was selected as the activation function. The mean square error (MSE) was used as the cost function because it gives more emphasis on reducing the larger errors. For the evaluation of the system, the dataset was randomly split into three subsets as follows: 65% training data, 15% validation data, and 20% test data. The purpose of the validation set was to fine-tune the hyperparameters and to prevent overfitting. The optimized parameters were the learning rate and the number of epochs. The simulation process was repeated five times in order to prove its effectiveness. Network training lasts from 12 to 45 epochs for best validation performance as shown in Figs. 9 and 10.

**Fig. 11:**
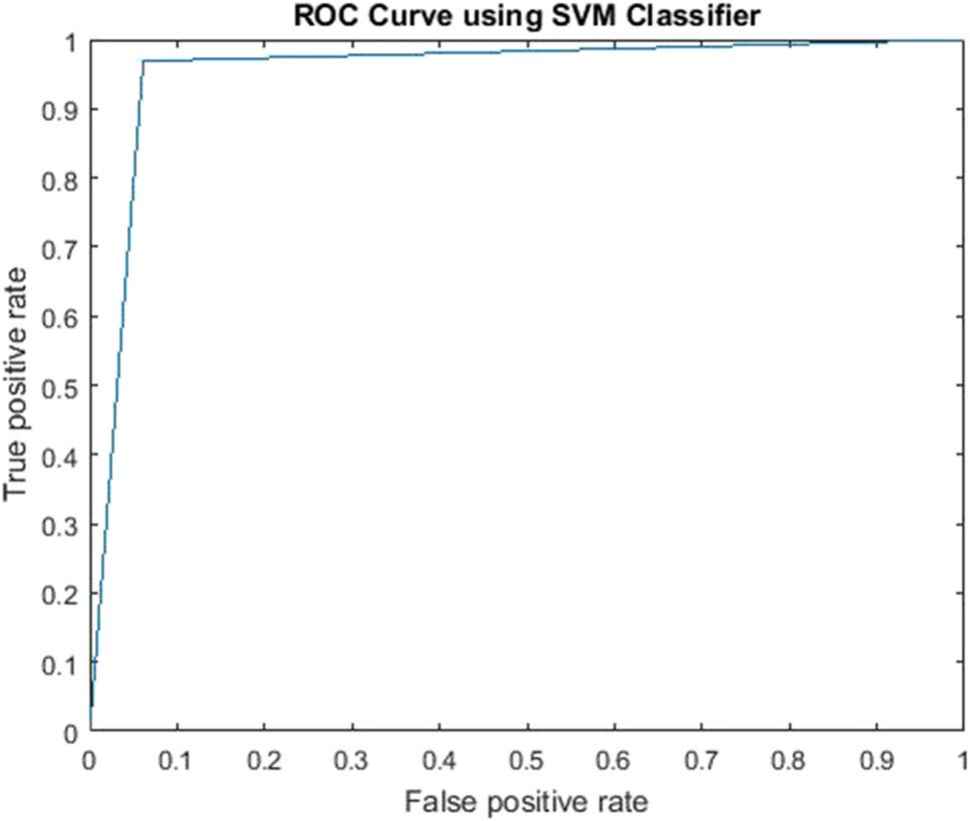
False positive rate using the SVM classifier

In the SVM classifier, a Gaussian kernel was used along with the SMOTE (synthetic minority oversampling technique) in order to make our dataset balanced [70]. This technique has also been used in other relevant studies [71]. The dataset was tested using fivefold cross-validation, dividing the dataset randomly into five roughly equal subsets/folds. Each fold was used as the validation set once, and the remaining fourfold was used as the training set. Therefore, the process was repeated five times, where each time a different fold was used as the validation data. The optimized hyperparameters were the kernel coefficient and the observation weights. The receiver operating characteristic curve is shown in Fig. 11. The area under curve value was calculated as 0.95.

## Results and Discussion

A CAD system can classify a region of interest as positive (lesion) or negative (normal breast tissue), and its decision can be either true (correct) or false (incorrect). The erroneous outputs are called false positive (FPs) and false negatives (FNs), whilst the correct decisions are true positives (TPs) and true negatives (TNs). The classification performance of the proposed CAD system was evaluated based on the following parameters:

- Accuracy: The ratio of the number of correct predictions to the total number of predictions.
- Sensitivity: The number of true positive predictions divided by the total number of the actual positive samples.
- Specificity: The proportion of the actual negative samples that are correctly identified.
- Precision: The ratio of the correctly predicted lesion samples to the total number of detected lesions.

All 138 lesions, 64 malignant and 74 benign, were correctly detected by the segmentation algorithm on the MRIs. According to the ranking results obtained by implementing the Relieff function, the texture parameters of homogeneity and energy could better differentiate the two classes.

As described in the previous section, we conducted the empirical evaluation of the proposed system using two alternative classifiers, a FFBPN and a SVM with Gaussian kernel function, whereas in the ANN different number of neurons were tested in the hidden layer. The performance results are presented in Table 1. The SVM model displayed the highest classification accuracy over the other models.

**Table 1:** Classification performance

Classifier	Accuracy	Sensitivity	Specificity	Precision
ANN (*n* = 6)	86.46%	83.31%	89.59%	88.71%
ANN (*n* = 10)	86.17%	83.12%	89.22%	88.37%
SVM	88.77%	92.25%	85.71%	85%

Table 2 presents sensitivity results of other similar studies that used traditional machine learning and deep learning techniques on DCE-MRI data. Even though these techniques were not tested on the same data, the relative merit of the proposed system can be highlighted based on the presented results. All results are taken from the original papers.

**Table 2:** Results of other similar studies that used traditional ML and deep learning techniques

Study	Classifier	Sensitivity
Zheng et al. [40]	Linear classifier	95%
Rasti et al. [41]	ME-CNN	97.73%
Shokouhi et al. [42]	SVM	94%
Hu et al. [43]	CNN + SVM	77.90%
Proposed model	SVM	92.25%

Furthermore, the two classifiers were additionally compared using the McNemar test. It is a common non-parametric test used in binary classification, and it typically compares either sensitivity or specificity of two classifiers [72]. For the test execution, the built-in MATLAB function testcholdout was used, which can conduct several McNemar test variations, including the asymptotic test, the exact-conditional test, and the mid-*p*-value test. The function first compares the predicted labels obtained by the two classification models against the true labels, and then detects whether the difference between the misclassification rates is statistically significant. The null hypothesis *H*_0_ claims that the two models have similar performance, whilst *H*_1_ indicates rejection of the null hypothesis at the 5% significance level. The test was repeated several times to draw reliable results, utilizing additional function parameters, such as the type of the conducted test, where the exact-conditional test and the mid-*p*-value test were carried out. Moreover, the parameter of the alternative hypothesis assessment was also utilized, where the altered null hypothesis claimed that the SVM classifier performed better than an ANN classifier. According to the conducted tests, the SVM model outperformed the ANN models. Examples of the obtained test results are presented in Table 3.

**Table 3:** McNemar test results where the alternative null hypothesis indicates that the SVM classifier performs better than the ANN classifiers having *n* = 6 and *n* = 10 neurons in the hidden layer. h and p denote the accepted hypothesis (*H*_0_ or *H*_1_) and the calculated *p*-value respectively

	Exact-conditional test	Mid-*p*-value test	Exact-conditional test	Mid-*p*-value test
	ANN with 6 neurons		ANN with 10 neurons	
*h*	0	0	0	0
*p*	0.8125	0.65625	0.5	0. 37695

The main advantages of the proposed system over the existing methods are, firstly, the novel breast region segmentation method which identifies automatically the approximate size and location of the breast tissue, in contrast to related studies where the breast region is segmented manually, or the rectangular cropping window is determined empirically based on the acquisition settings [41, 46]. Furthermore, the cropping window is calculated using the intensity values of the image pixels, combined with image rotations, avoiding the use of complex algorithms that negatively affect the system’s complexity [42]. Secondly, the ability of the proposed segmentation algorithm to successfully detect and segment the suspicious regions of interest (ROIs), considering the dynamic enhancement changes across only two post-contrast sequential phases. The approach in which all post-contrast phases are considered complicates processing and may result in a bias of benign over malignant lesions in the obtained dataset, since in the delayed post-contrast subtraction images most malignant lesions are not identifiable because of rapid wash-out, whilst benign lesions continue to enhance due to slow wash-in. In other similar studies, ROIs are identified using the image obtained before the administration of the contrast agent, or with the additional contribution of only one post-contrast image [16, 41, 58]. Moreover, there are studies in which ROIs are identified manually by an experienced radiologist [50] or by using a region growing technique following manual seeding [36]. Thirdly, most of the medical imaging datasets have imbalanced class samples which may impact the prediction performance [21, 35]. Hence, to overcome this issue, the SMOTE was used. Additionally, the novel approach of the control ROIs group creation allows the computation of a set of features that sufficiently discriminate lesions from healthy breast tissue without the need of additional data, which is a common problem in single-centre research studies. A similar approach was presented in the work of Ribli et al. [35] where each side of the breasts was treated as separate cases. Lastly, patients’ age covers a wide age range proving that the system’s performance is not affected by the patients’ breast density, since according to literature there is an inverse relationship between women age and breast density [73, 74].

There are several limitations in our study. Firstly, this was a single-centre study, and the sample size was relatively small. In future work, further evaluation of the system needs to be performed on a larger dataset and with more models than those employed in this study, which present specific drawbacks. Secondly, even though the developed CAD system is able to detect all the lesions existing in the DCE-MRI images, in some cases, their boundaries or sizes differ from the ones recorded in the available radiologists’ reports. However, as several studies mention, the detection of a lesion is more important than to accurately find its limits. Furthermore, CAD systems are not able to replace an experienced radiologist, but they are considered a second opinion helping them to identify lesions that were mistakenly misinterpreted [75]. Thirdly, the radiomic approach adopted in this study was solely based on the GLCM matrix and further studies should exploit the wide range of applicable features and filters for better highlighting regional patterns. Finally, although the proposed system exhibited high performance, we must point out that the objective of this study was only lesion detection. Discrimination between malignant and benign lesions should be further explored in a future study, adding morphological and kinetic features to the calculated feature vector. For the use of kinetic features, a registration step needs to be added in the existing workflow, registering all post-contrast sequences to the pre-contrast sequence, before lesion detection. Then, the kinetic features for each detected lesion would be extracted from the corresponding time-intensity curve following appropriate processing. A further goal would be to incorporate deep learning methods in the proposed methodology, since they have been extensively used in recent studies of medical imaging. In particular, deep learning techniques could be used in the feature extraction step, avoiding the use of handcrafted features, as well as in the classification step, provided that the examined dataset is large enough.

## Conclusions

In this study, an automated CAD system is proposed for detecting breast lesions in DCE-MRI. The contribution of the CAD system is to detect potential malignant or benign lesion regions using a multilevel thresholding technique considering the dynamic enhancement changes across two post-contrast sequential phases. Moreover, a novel breast region segmentation method is introduced, which identifies and crops the breast tissue, reducing the processed image region and, thus, resulting in relatively lower computational burden compared to the situation in which the whole image is processed. For the classification stage, we propose the application of a two-stage false positive reduction process to distinguish better lesions from healthy breast tissue. The experimental results on our dataset of 119 DCE-MRI slices, obtained from 52 DCE-MRI examinations, have shown that the proposed CAD achieves high performance in identifying enhancing lesions and in differentiating them from healthy parenchyma. Future research will aim at evaluating the proposed CAD system over larger datasets as well as enriching our model with deep learning methods.

## Data Availability

The datasets analyzed during the current retrospective study are not publicly available due to patient privacy.
